# A Network Biology Approach to Discover the Molecular Biomarker Associated with Hepatocellular Carcinoma

**DOI:** 10.1155/2014/278956

**Published:** 2014-05-14

**Authors:** Liwei Zhuang, Yun Wu, Jiwu Han, Xiaohua Ling, Liguo Wang, Chengyan Zhu, Yili Fu

**Affiliations:** ^1^State Key Laboratory of Robotics and System, Bio-X Centre, Harbin Institute of Technology, Harbin, Heilongjiang 150001, China; ^2^Department of Gastroenterology, The Fourth Affiliated Hospital of Harbin Medical University, Harbin, Heilongjiang 150001, China

## Abstract

In recent years, high throughput technologies such as microarray platform have provided a new avenue for hepatocellular carcinoma (HCC) investigation. Traditionally, gene sets enrichment analysis of survival related genes is commonly used to reveal the underlying functional mechanisms. However, this approach usually produces too many candidate genes and cannot discover detailed signaling transduction cascades, which greatly limits their clinical application such as biomarker development. In this study, we have proposed a network biology approach to discover novel biomarkers from multidimensional omics data. This approach effectively combines clinical survival data with topological characteristics of human protein interaction networks and patients expression profiling data. It can produce novel network based biomarkers together with biological understanding of molecular mechanism. We have analyzed eighty HCC expression profiling arrays and identified that extracellular matrix and programmed cell death are the main themes related to HCC progression. Compared with traditional enrichment analysis, this approach can provide concrete and testable hypothesis on functional mechanism. Furthermore, the identified subnetworks can potentially be used as suitable targets for therapeutic intervention in HCC.

## 1. Introduction

Liver cancer is one of the leading malignancies of cancer-related deaths worldwide [1]. Hepatocellular carcinoma (HCC), which accounts for about 85% of the primary liver cancer cases, has been associated with a variety of risk factors including chronic viral hepatitis B and C infections, alcohol abuse, autoimmune hepatitis, primary biliary cirrhosis, and nonalcoholic steatohepatitis [2]. Since HCC is difficult to be detected at its early stage, the 5-year survival rate is only about 44% [3]. Surgery and other palliative treatments including chemotherapy, transarterial embolization, and radiotherapy are the standard treatments for HCC. Unfortunately, these adjuvant therapies have only a modest impact on survival time. This situation indicates that development of sensitive diagnostic biomarker used in the early stage of HCC will greatly lead to improved survival of patients.

Previous investigations have shown that HCC is fundamentally a heterogenetic disease and multiple signaling pathways contribute to HCC progression [4]. Therefore, a systematic assessment of the functional network in which these genes interconnect may lead to a more precise set of alterations which could be served as key biomarkers or drug targets for clinical interrogation. In recent years, high throughput technologies such as microarray platform and large scale of protein-protein interaction (PPI) discovery have provided a new avenue for biomarker development of HCC [5–7]. In this study, we have adopted an integrative approach to identify network based biomarker from these omics data. We used a multivariate Cox proportional hazards model to quantify the correlation between the expression profiles of survival gene groups and patient survival data. These gene groups were preselected according to PPI network structure. This approach can produce novel network based biomarkers together with biological understanding of molecular mechanism. We have analyzed eighty HCC expression profiling arrays and identified that extracellular matrix (ECM) and programmed cell death are the main themes related to HCC survival data. Based on manual survey of publications, we found that several previously implicated genes with clinical significance were contained in these two subnetworks. Compared with Gene Ontology enrichment analysis, our approach can provide concise functional mechanism hypothesis and is useful for biomarker development.

## 2. Materials and Methods

### 2.1. Datasets

The gene expression data and the corresponding clinical data were downloaded from NCBI Gene Expression Omnibus (GEO) database (http://www.ncbi.nlm.nih.gov/geo/query/acc.cgi?acc=GSE10141). Genome-wide expression profiling of formalin-fixed, paraffin-embedded tissues, which are from 80 HCC cancer patients, was measured in Human 6k Transcriptionally Informative Gene Panel for DASL microarray platform. For multiple probes for a particular gene, we calculated its signal intensity as the mean of intensities of all these probe sets in this sample. Robust Multiarray Average (RMA) was used to normalize signal intensity within each dataset. The normalized expression values were used in follow-up analysis.

The protein-protein interactions data from Human Protein Reference Database (HPRD, http://hprd.org/) was used in this study. Currently, HPRD contains manually curated over 42,000 interactions between 7514 human genes.

### 2.2. Identification of Survival Related Subnetworks

In the human protein-protein network, each node (protein) with corresponding gene expression value was regarded as “seed node.” For a seed node *i*, this node and its neighbours *j* within the shortest distance *k* form a connected subnetwork with *n* nodes [8]. A multivariate Cox proportional hazards regression model was used to quantify the correlation between the expression profiles of *n* genes in each subnetwork and patient survival data. The Wald *χ* ^2^ test was used to determine the significance of each predictor's hazard toward the survival time, with the overall survival months as the dependent variable [9]. The multivariable Cox *P* values are adjusted by false discovery rate correction. The searching starts from a seeded gene *i* and finds all subnetworksaccording to *k*. Since our aim is to discover clinically applicable biomarkers from PPI network data, therefore, we should control the size of obtained networks while providing testable experimental hypothesis at the same time. Considering this practical situation, we added a constraint to reduce the searching space and set the the shortest distance as 3; that is, the shortest distance between seed gene *i* and its neighbours *j* is smaller than or equal to 3 (*k* ≤ 3). Then, all the subnetworks that fulfilled the above criterion were evaluated in a multivariate Cox model [9]. The subnetwork with minimum *P* value was reported for that seeded gene. All the above computations were conducted in *R* statistical package (http://www.r-project.org/).

### 2.3. Gene Sets Enrichment Analysis

In order to assess the results, we also used a univariate Cox proportional hazards model to correlate each individual gene expression data with survival data (at *P* < 0.05 level). This computation was done on all genes to genome-wide select candidate survival related genes. Gene sets enrichment analysis of candidate gene list is a commonly used statistical technique to reveal the underlying functional mechanisms based on large collections of functional annotations. Many bioinformatics tools have been developed for this purpose. In this study, we used ToppGene Suite (http://toppgene.cchmc.org/) to correlate the survival genes generated by a univariate Cox model with Gene Ontology functional annotations. Multiple statistical tests were controlled by false discovery rate at 0.05 level.

## 3. Results 

To identify network based biomarker in HCC and its potential mechanism related to cancer progression, we first mapped each node in the protein-protein network to HCC profiling data; the nodes with expression values were regarded as “seed nodes.” Each “seed node” together with its neighbours*j* within the shortest distance *k* forms a gene group. Next, we used a multivariate Cox proportional hazards model to quantify the correlation between the expression profiles of the gene group and patient survival data. All the subnetworks within a gene group were evaluated with the stepwise variable selection procedure in a multivariate Cox model [9]. The subnetwork with the minimum multivariate Cox *P* value was reported for that “seed gene.” Note that the genes within a group were constrained by the PPI network topological structure (i.e., connectivity between nodes).

We combined human PPI data from HPRD database and a set of gene expression data for fixed HCC tissues (GSE10141) to test our computational framework. Among the 7514 nodes (genes) in PPI network, 3371 nodes can be mapped to HCC profiling data. With an adjusted multivariable Cox *P* value 0.001, we totally get 11 compact survival related subnetworks. The average number of genes in each survival related subnetwork was 8.6 and the average number of interactions (edge) is 7.8 (see Supplementary File 1, available online at http://dx.doi.org/10.1155/2014/278956). 

**Figure 1:**
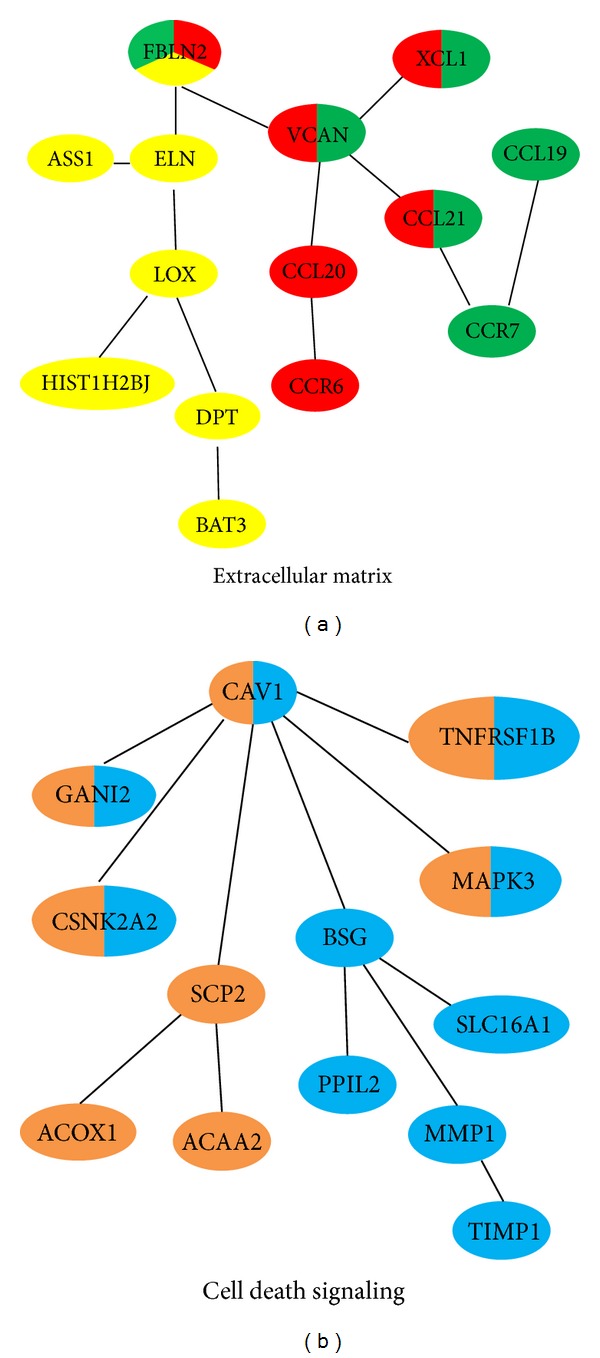
Survival related subnetworks. (a) and (b) indicated, respectively, extracellular matrix and cell death signaling modules correlated survival time in liver cancer. The top 5 ranked survival related subnetworks are labelled with different colors. CCR6-CCL20 subnetwork is labelled in red; CCR7-CCL21 subnetwork is labelled in green; BAT3 subnetwork is labelled in yellow; BSG subnetwork is labelled in blue; SCP2 subnetwork is labelled in brown. Note that some nodes (proteins) with more than one color mean that these proteins are involved in more than one top ranked survival related subnetwork.

The top 5 significant subnetworks are summarized in Table 1. Inspecting this list, we found that these five subnetworks can be classified into two larger functional modules as illustrated in Figure 1. We called the first one “extracellular matrix (ECM) module” since most of the genes in this module have been associated with ECM (Figure 1(a)). This module consists of CCR6- (chemokine receptor 6-) CCL20 (chemokine ligand 20), CCR7- (chemokine receptor-) CCL21 (chemokine ligand 21), and BAT3 (also known as BCL2-associated athanogene 6) subnetworks. CCR6-CCL20 (the top 4 subnetworks) and CCR7-CCL21 (the top 1 subnetwork) form the chemokines signaling branch of ECM module. Chemokines are a family of small signaling molecules, which contain a structural homologous conservative family of cysteine residues. Previous studies have demonstrated that chemokines and their receptors play a critical role in HCC progression [10, 11]. In particular, the expression of CCL20/CCR6 is highly increased in HCC tissues of grade III tumors in comparison to grade II tumors [12].

Both of the two chemokines signaling subnetworks converge to FBLN2 (fibulin 2) (Figure 1(a)). FBLN2 is an extracellular matrix protein, which belongs to the fibulin family. This protein binds various extracellular ligands and calcium. Recent study indicates that FBLN2 may play a role during progression in a variety of cancers. For example, FBLN2 was found dramatically downregulated in NPC (nasopharyngeal carcinoma) and overexpression of FBLN2 inhibits cancer cell proliferation, migration, invasion, and angiogenesis* in vitro *[13]. In another report, FBLN2 locus was high methylated in breast, childhood acute lymphoblastic leukemia, and other common epithelial cancers (lung, colorectal, and prostate) [14, 15].

FBLN2 is connected with BAT3 branch (the top 2 subnetworks) including ELN (elastin), ASS1 (argininosuccinate synthase 1), LOX (lysyl oxidase), HIST1H2BJ (histone cluster 1, H2bj), and DPT (dermatopontin) (Figure 1(a)). BAT3 is a nuclear protein that is cleaved by caspase 3 and is implicated in the control of apoptosis. In addition, the protein forms a complex with E1A binding protein p300 and is required for the acetylation of p53 in response to DNA damage.

The top 3 and top 5 subnetworks form a large module around CAV1 (caveolin 1) (Figure 1(b)). This scaffolding protein is a tumor suppressor gene candidate and a negative regulator of the apoptosis cascade. This signaling complex includes the BSG (basigin) branch and the SCP2 (sterol carrier protein 2) branch. Accumulated evidences indicated that the BSG branch is involved in programmed cell death. For example, BSG, a tumor-related glycoprotein, is highly expressed in hepatocellular carcinoma cells and fibroblasts. Recently, it was found that BSG can mediate both apoptosis and autophagy, two main cell death patterns in human hepatoma cells [16, 17]. SCP2 is highly expressed in liver and involved in the transportation of common phospholipids, cholesterol, and gangliosides between membranes. Both the SCP2 and BSG branches share the same CAV1 upstream regulators such as MAPK3, TNFRSF1B, and GNAI2, which have been implicated in cancer cells death pathways [18–20]. Importantly, CAV1 was found to negatively regulate TRAIL-induced apoptosis in human hepatocarcinoma cells [21]. For example, Yang et al. also demonstrate that overexpression of CAV1 can increase the cytotoxic and proapoptotic activity of resveratrol in a dose- and time-dependent manner in a hepatocellular carcinoma animal model [22]. Similarly, CAV1 was found to be exclusively expressed in HCC cell lines and tissues [23]. CAV1 overexpression was significantly correlated with the invasive and metastatic ability of HCC [24]. Thus, from the above analysis, CAV1 may mediate the crosstalk between liver metabolism and cell death signaling pathway and is a potential biomarker in clinical practice.

### 3.1. Gene Ontology Enrichment Analysis Demonstrates the Validity of the Survival Network

As a comparison, we also conduct the enrichment analysis of survival correlated gene (hereafter referred to as survival genes) to Gene Ontology (GO). GO categories can be classified into the following 3 functional categories: (1) cellular component; (2) biological process; (3) molecular function. First, using a univariate Cox proportional hazards model, 336 genes were found significantly correlated with patients' survival data (at *P* < 0.05 level). These survival correlated genes were listed in Supplementary Table 2. With a cutoff of FDR < 0.05, we identified 7 GO cellular component gene sets, 217 biological process gene sets, and 2 molecular function gene sets that are enriched with survival gene (Supplementary Table 3). The top 5 significant GO terms in each functional category are summarized in Table 2. From the viewpoint of molecular function, the top ranked GO terms include “receptor binding” and “protein complex binding.” From the viewpoint of cellular component, the top ranked GO terms include “integral to/intrinsic to plasma membrane” and “extracellular region part.” All the above results clearly reflect the molecular changes at extracellular matrix region. On the other hand, the programmed cell death function is dominated in the selected GO biological process categories (Table 2). Therefore, both ECM and programmed cell death, the two master themes identified in our network analysis, were reproduced from GO analysis.

Although GO analysis demonstrates the validity of our network based procedure, our analysis outperforms GO analysis. First, the problem with traditional GO enrichment is that each identified gene set usually includes too many genes, which greatly limits their clinical application. For instance, in the above GO enrichment data there are over 60 and 23 survival genes that are enriched within GO term cell death and ECM (Supplementary Table 3). On the other hand, in our network analysis results, there are only 13 and 14 potential biomarker genes in cell death and ECM, respectively (Supplementary Table 1). More importantly, our results can also simultaneously provide the interaction relationships among these candidate proteins, which will provide direct mechanism understanding of HCC progression and greatly facilitated further experimental verification. In comparison, the signaling transduction cascades between candidate genes in the identified GO categories are elusive.

## 4. Discussion

HCC accounts for over 85% of liver cancer cases and is a lethal malignancy with high mortality rates. However, better outcomes have been observed for tumors detected at an earlier stage. This clearly indicates that detecting the HCC at earlier stage can significantly benefit the HCC patients. In recent years, although a wide range of molecular biomarkers, including Glypican-3 [25], GP73 [26], and other oxidative stress related biomarkers [27], have been developed, most of them lack adequate functional significance with HCC. Thus, how those findings could be applied in daily clinical practice remains unknown. Recently, the large scale omics data present both significant challenges and opportunities for improving our understanding and treatment of this highly aggressive and lethal disease. We have adopted an integrative approach to prioritize genes of potential importance in HCC.

Our network based approach involved multidimensional analysis of gene expression, PPI network, and clinical data. This novel strategy allows us to successfully discover five ECM and cell death signaling related subnetworks as survival subnetworks in HCC. Importantly, our approach can decipher the detailed molecular mechanism among genes. For example, recently, interests are rising to detect the role of ECM in cancer progression [28, 29]. ECM protein could be dynamically regulated by either tumour cells or tumour stromal components from the tumor microenvironment. After initiation, this family of proteins can transduce intracellular downstream signalling events that lead to cell death, invasion, and matrix remodeling. But how matrix integrity can mediate the signaling transduction from the extracellular region to the inner cell is largely undefined. In our ECM module, the two chemokines signaling subnetworks can sense the stress from environment, and then the signals can be transduced to internal response such as apoptosis via FBLN2 and nuclear effector such as BAT3. Thus, we speculate that this route may be one of the stress signaling pathways used in liver cells.

Similarly, a number of studies have showed that the CCL20-CCR6 axis is strongly associated with HCC. For example, Rubie et al. [12] found that CCL20 was the only chemokine that showed significant overexpression in HCC tissues. Fujii et al. [10] also found that CCL20-CCR6 axis promotes Huh7 hepatoma cell growth* in vivo*. More importantly, clinicopathological analysis revealed that the incidence of intrahepatic metastasis was higher in patients with increased expression of CCR6 compared to patients with low expression of CCR6. Furthermore, disease-free survival was significantly poorer in the patient groups with high CCR6 expression [30]. All of these data are consistent with our results and indicate that CCL20-CCR6 axis is a promising biomarker for HCC.

Our results provided testable hypothesis for the exact mechanism by which the CCL20-CCR6 axis inhibits the apoptosis and promotes the cancer growth. Specifically, we showed that CCR and their ligands may contribute to cancer progression in part via the FBLN2-BAT3 branch of the cell death module (Figure 1(a)).

Note that our network based approach can efficiently detect the synergistic effects among the included genes in subnetworks. As a comparison, we also computed the univariate Cox *P* value for each of the genes in subnetwork (Table 1). It is clear that some of the genes are actually not significantly solely based on a univariate Cox *P* value. But we can also see that the *P* value of the multivariate Cox model is lower than any of the univariate Cox *P* values when the genes within one subnetwork were assessed together. This phenomenon clearly demonstrates the power of network biology method in detecting systemic changes, which are commonly seen in living cells.

Our approach offers a paradigm for future use of network biology concept and method for therapeutic intervention in HCC. Although our study is a preliminary analysis of HCC and needs further verification, it provides a novel avenue to develop new generation of network based biomarkers in HCC.

## 5. Conclusion

A multivariate Cox model was used to identify network biomarker based on HCC expression profiling and PPI. CAV1 induced cell death subnetwork and ECM subnetwork were identified as putative clinical network based biomarkers and possible targets of individualized therapy in HCC. These results provided new insights into understanding the potential mechanisms that govern the HCC progression.

## Supplementary Material

Supplementary File 1: contains the identified survival related subnetworks. For each subnetworks, the seed node, the component genes, the number of nodes and the the number of edges are reported.
Supplementary File 2: contains survival gene list selected by univariate cox proportionhazards model. The gene symbol,name,accession number and univariate cox p-value are reported.
Supplementary File 3: contains the Gene Ontology categories selected by enrichment analysis of survival gene. For each category, the GO ID, name,FDR and number of total genes are reported.

## Figures and Tables

**Table 1:** Top five ranked survival related subnetworks.

Network rank	Component genes	Univariate Cox *P* value	Adjusted multivariable Cox *P* values
1	CCR7	3.10*E* − 01	8.69*E* − 06
	XCL1	5.10*E* − 03	
	VCAN	2.00*E* − 02	
	CCL21	4.80*E* − 03	
	CCL19	4.80*E* − 03	
	FBLN2	5.00*E* − 04	

2	HIST1H2BJ	2.60*E* − 01	1.02*E* − 05
	LOX	1.70*E* − 01	
	DPT	3.00*E* − 02	
	BAT3	5.30*E* − 02	
	ELN	4.70*E* − 02	
	FBLN2	5.00*E* − 04	
	ASS1	5.10*E* − 02	

3	PPIL2	2.30*E* − 01	1.61*E* − 05
	BSG	1.90*E* − 01	
	MMP1	2.00*E* − 04	
	SLC16A1	6.00*E* − 02	
	TIMP1	1.50*E* − 01	
	CAV1	1.30*E* − 01	
	TNFRSF1B	2.70*E* − 03	
	CSNK2A2	3.50*E* − 02	
	GNAI2	3.20*E* − 02	
	MAPK3	3.30*E* − 02	

4	CCR6	4.50*E* − 01	4.59*E* − 05
	CCL20	2.10*E* − 01	
	VCAN	2.00*E* − 02	
	FBLN2	4.00*E* − 04	
	CCL21	4.80*E* − 03	
	XCL1	5.10*E* − 03	

5	ACAA2	2.60*E* − 02	9.65*E* − 05
	SCP2	1.30*E* − 01	
	ACOX1	8.20*E* − 01	
	CAV1	1.30*E* − 01	
	TNFRSF1B	2.70*E* − 03	
	CSNK2A2	3.50*E* − 02	
	GNAI2	3.20*E* − 02	
	MAPK3	3.30*E* − 02	

**Table 2:** Top five significant GO categories that are enriched with survival genes.

Category rank	Cellular component	FDR	Biological process	FDR	Molecular function	FDR
1	GO:0005887: integral to plasma membrane	3.92*E* − 04	GO:0043067: regulation of programmed cell death	1.41*E* − 05	GO:0005102: receptor binding	1.51*E* − 02
2	GO:0031226: intrinsic to plasma membrane	3.92*E* − 04	GO:0060548: negative regulation of cell death	3.22*E* − 05	GO:0032403: protein complex binding	4.04*E* − 02
3	GO:0044421: extracellular region part	2.53*E* − 03	GO:0042981: regulation of apoptotic process	3.22*E* − 05		
4	GO:0031012: extracellular matrix	4.87*E* − 03	GO:2000145 regulation of cell motility	7.28*E* − 05		
5	GO:0009986: cell surface	1.50*E* − 02	GO:0050863 regulation of T cell activation	9.26*E* − 05		
